# The GTPase ARF6 Controls ROS Production to Mediate Angiotensin II-Induced Vascular Smooth Muscle Cell Proliferation

**DOI:** 10.1371/journal.pone.0148097

**Published:** 2016-01-29

**Authors:** Mohamed Bourmoum, Ricardo Charles, Audrey Claing

**Affiliations:** Department of Pharmacology, Faculty of Medicine, Université de Montréal, Montreal, Quebec, H3T 1J4, Canada; Albany Medical College, UNITED STATES

## Abstract

High reactive oxygen species (ROS) levels and enhanced vascular smooth muscle cells (VSMC) proliferation are observed in numerous cardiovascular diseases. The mechanisms by which hormones such as angiotensin II (Ang II) acts to promote these cellular responses remain poorly understood. We have previously shown that the ADP-ribosylation factor 6 (ARF6), a molecular switch that coordinates intracellular signaling events can be activated by the Ang II receptor (AT_1_R). Whether this small GTP-binding protein controls the signaling events leading to ROS production and therefore Ang II-dependent VSMC proliferation, remains however unknown. Here, we demonstrate that in rat aortic VSMC, Ang II stimulation led to the subsequent activation of ARF6 and Rac1, a key regulator of NADPH oxidase activity. Using RNA interference, we showed that ARF6 is essential for ROS generation since in conditions where this GTPase was knocked down, Ang II could no longer promote superoxide anion production. In addition to regulating Rac1 activity, ARF6 also controlled expression of the NADPH oxidase 1 (Nox 1) as well as the ability of the EGFR to become transactivated. Finally, ARF6 also controlled MAPK (Erk1/2, p38 and Jnk) activation, a key pathway of VSMC proliferation. Altogether, our findings demonstrate that Ang II promotes activation of ARF6 to controls ROS production by regulating Rac1 activation and Nox1 expression. In turn, increased ROS acts to activate the MAPK pathway. These signaling events represent a new molecular mechanism by which Ang II can promote proliferation of VSMC.

## Introduction

Vascular smooth muscle cells (VSMC) proliferation is a crucial event during normal vascular development. However, this cellular process plays a major role in cardiovascular diseases such as atherosclerosis, restenosis after angioplasty and hypertension [1–3]. Among all hormones and growth factors, angiotensin II (Ang II) signaling pathways have been widely studied in this context [4–6]. Most of the physiological and pathophysiological actions of this 8-amino acid peptide are mediated by the activation of its best-characterized receptor: the angiotensin II type 1 receptor (AT_1_R). This membrane protein, which couples to heterotrimeric G protein complexes, promotes the activation of classical downstream effectors including phospholipase C (PLC), phospholipase A_2_ (PLA_2_), and phospholipase D (PLD) [7, 8]. AT_1_R activation can also mediate signaling via ßarrestin recruitment or transactivation of tyrosine kinase receptors (RTK). These often lead to the activation of mitogenic signaling (Erk, p38, Jnk) [9].

Reactive oxygen species (ROS) have also been shown to function as important signaling molecules in promoting VSMC proliferation [10, 11]. ROS can be generated by a number of pathways including NADPH oxidase (Nox) enzymes. Aortic VSMC express Nox1 and Nox4 in rodents as well as Nox5 in humans [12] and Ang II stimulation has been reported to increase intracellular levels of ROS although the mechanism by which it does has yet to be fully elucidated. Previous studies have reported that enzymes such as PLD, PKC, Src and PI3Kß may play a role [13, 14]. Interestingly, activation of Rac1, a small GTP-binding protein of the Rho family, in addition to its recruitment to Nox1, are crucial events for superoxide anion production following Ang II stimulation of VSMC [15]. Furthermore, transactivation of the epidermal growth factor receptor (EGFR) was reported to involve activation of the metalloprotease ADAM17, shedding of HB-EGF and ROS production [16]. Finally, it was suggested that activation of MAPK was sensitive to ROS. For example, Jnk and p38 activation, in response to Ang II, can be blocked by several antioxidants [17, 18], while the sensitivity of Erk1/2 remains controversial [18–21].

Our previous work has brought attention to another family of small GTP-binding proteins as key molecular switches activated by the AT_1_R: the ADP-ribosylation factors (ARF). These were initially characterized as key molecules regulating vesicle trafficking [22, 23]. Six members of this Ras-related family of proteins have been identified. The two best-characterized isoforms are ARF1 and ARF6. In HEK 293 cells stably expressing the AT_1_R, we reported that Ang II stimulation results in the activation of ARF6, an isoform associated with the plasma membrane. This GTPase is known to regulate receptor endocytosis and actin remodeling [24]. In addition, ARF6 activation, by this receptor, controls activation of Rac1, another GTPase. Like all GTP-binding proteins, ARF6 cycles between its inactive (GDP-bound) and active (GTP-bound) form. This cycling is controlled by guanine nucleotide exchange factors (GEF) and GTPase-activating proteins (GAP). ARF6 has been associated with proliferation in many cell lines; overexpression of the dominant negative mutant ARF6T^27^N was effective in reducing VEGF-induced proliferation of endothelial cells [25]. Numerous studies suggest that ARF6 is implicated in the proliferation of cancer cells [26, 27]. Furthermore, cross talk between ARF6 and Rac1 was demonstrated in many cellular functions. First, ARF6 mediates peripheral actin rearrangement through Arfaptin2, a Rac1-interacting protein [28]. Coordinated action of ARF6 and Rac1 was also reported in neurite outgrowth and epithelial cell scattering [29–31] and we showed that ARF6 can interact with Rac1 upon Ang II stimulation to regulate membrane ruffling and cellular migration [24].

Because of the increasing interest in understanding the role of ROS in cardiovascular diseases, we aimed here at examining whether ARF6 could regulate ROS production and ultimately proliferation of VSMC. Our findings demonstrate that besides its traditional role in endocytosis and actin remodelling, ARF6 is a pivotal player in Ang II signaling. We show for the first time that this GTPase regulates ROS generation by controlling Rac1 activation and Nox1 expression. The ability of ARF6 to control Rac activity directly impacts the ability of this GTPase to control, in turn, the function of Nox1, the main ROS generating enzyme in our cells. In addition, by acting at the level of Nox1 gene expression, ARF6 can directly impact ROS production. Through ROS, ARF6 also regulates EGFR transactivation and MAPK activation. Activation of these key signaling events are important for cellular responses such as proliferation. In this study, we show that depletion of ARF6, expression of a Rac1 dominant negative mutant or treatment of the cells with EGFR as well as ROS inhibitors greatly impairs the ability of Ang II to promote VSMC proliferation. Altogether, our findings demonstrate that the GTPase ARF6 acts as a molecular switch to activate Ang II-mediated signaling pathways leading to ROS production and MAPK activation, to regulate VSMC proliferation.

## Materials and Methods

### Reagents

Lipofectamine 2000^TM^ was purchased from Invitrogen (Burlington, ON, Canada). Ang II, Dihydroethidium (DHE), diphenyliodonium (DPI), Thiazolyl Blue Tetrazolium Bromide, cytochrome C, ML171 and AG1478 were purchased from Sigma Aldrich (Oakville, ON, Canada). Antibodies against ARF6, HA-tag, Erk1/2, Noxa1 and Noxo1 were from Santa Cruz Biotech (Santa Cruz, CA, USA). Anti-Rac1 and Myc-tag monoclonal antibodies were from Millipore Corporation (Mississauga, ON, Canada). Antibodies against actin, phospho-Erk1/2, p38, phospho-p38, Jnk, phospho-Jnk, EGFR, phospho-Tyr^1086^ EGFR and phosphoTyr^845^ EGFR were purchased from Cell Signaling (Danvers, MA, USA). Anti-Nox1 was from Bioss Antibodies (Woburn, MA, USA). Anti-Nox4 was from Abcam (Toronto, ON, Canada). Goat anti-mouse and goat anti-rabbit horseradish peroxidase conjugated antibodies were from R&D Systems (Minneapolis, MN, USA). ARF6 and Rac1 shRNA were obtained as lentiviral plasmids (pLKO.1-puro) provided as bacterial glycerol stocks by Sigma Aldrich (Oakville, ON, Canada). Lentiviruses were then produced in HEK293T cells using an adapted protocol from Laurie Ailles/ Weissman Lab (Mississauga, Ontario, Canada) and Clontech protocols (Clontech Laboratories, Inc). Sequences of shARF6 and shRac1 are found in MISSION® shRNA Library, Sigma Aldrich (ARF6 shRNA, Clone ID: NM_001663.3-926s21c1, sequence: ACCGGAGCTGCACCGCATTATCAATGCTCGAGCATTGATAATGCGGTGCAGCTTTTT TTG. Rac1 shRNA, Clone ID: NM_009007.2–544 s21c1, Sequence: CCGGGCTTGATCTT AGGGATGATAACTCGAGTTAT CATCCCTAAGATCAAGCTTTTTG.

### Cell culture, transfection and lentiviral transduction

All experiments were carried out using rat aortic VSMC clone SV40LT-SMC purchased from ATCC (Manassas, VA, USA). Cells were maintained in DMEM supplemented with 10% fetal bovine serum and penicillin/streptomycin (Wisent, QC, Canada) and incubated at 37°C in a humidified atmosphere of 95% air and 5% CO_2_. For ARF6 and Rac1 constructs overexpression, we transfected cells using Lipofectamine 2000 according to the manufacturer’s instructions. Cells were transfected with the empty or the ARF6/Rac1 DNA clones encoding vectors for 24h before being used for experiments. In ARF6 and Rac1 knock down experiments, we infected cells with the scrambled or the specific shRNA lentiviruses and media was changed after 8h. After 72h of lentiviruses infection, the targeted proteins were 100% depleted. Before all experiments, cells were serum starved for 48h (0.2% FBS).

### Western blotting

Cells were harvested in lysis buffer (50 mM Tris-HCl, 1% NP-40, 10% glycerol, 140 mM NaCl, 5 mM MgCl_2_, 20 mM NaF, 1 mM NaPPi and 1mM orthovanadate, pH 7.4) complemented with protease inhibitors aprotinin (5μg/ml), benzamidine (150 μg/ml), leupeptin (5 μg/ml), pepstatin (4 μg/ml) and phenylmethylsulfonyl fluoride (1 mM). Cell lysates were solubilized at 4°C for 30 min and total soluble proteins were run on polyacrylamide gels and transferred onto nitrocellulose membranes. The membranes were blotted for relevant proteins using specific antibodies. Secondary antibodies were all horseradish peroxidase-conjugated. Protein expression was detected by chemiluminescence (ECL^TM^ Prime, GE Healthcare, Mississauga, ON, Canada). The digital images obtained were quantified using ImageJ software.

### GTPases activation assays

Activation levels of ARF6 and Rac1 were assessed using GST-pull down assays using GST-GGA3 and GST-PAK(CRIB) coupled to glutathione-sepharose 4B beads, respectively, as in [24]. ARF6-GTP and Rac1-GTP levels were detected by Western blot analysis.

### ROS measurement

DHE was used to evaluate superoxide anion levels. VSMC were seeded onto coverslips, left overnight, and serum starved for 48h (0.2% FBS). After Ang II stimulation (100 nM) for the indicated times, cells were washed twice with PBS and incubated with DHE (5 μM in HBSS) at 37°C for 15 min, in the dark. Cells were then washed, mounted onto slides, and observed by fluorescence microscopy (Zeiss, Germany) (excitation 535 λ, emission 595 λ). Images were taken and fluorescence quantification was performed using ImageJ software (3 to 4 images per slide representative of 1000–1500 cells per condition). For the cytochrome C reduction assay, seeded cells were serum starved for 48h (0.2% FBS) and stimulated or not with Ang II (100 nM, 60 min). The medium was then replaced by a buffer (145 mM NaCl, 4.8 mM KCl, 5.7 mM NaH_2_PO_4_, 0.54 mM CaCl_2_, 1.22 mM MgS0_4_, 5.5 mM glucose, 0.1 mM deferoxamine mesylate) containing 50 μM of acetylated cytochrome C and 1U/μl of catalase (to prevent re-oxidation of reduced cytochrome C by H_2_O_2_). Identical samples are incubated in the presence of 1U/μl of superoxide dismutase (SOD). Cells were then incubated at 37°C for 1h, in the dark. 200 μl post-incubation supernatants were transferred to a 96-well plate and absorbance (OD) was assessed at 540, 550 and 560 nm. Absorbance (optical density) of reduced cytochrome C was calculated using the formula: OD_550nm_—((OD_540nm_ +OD_560nm_/2). ODs from identical samples containing SOD were subtracted and only the SOD inhibitable value was considered.

### RNA extraction and quantification

Seeded control and ARF6-depleted VSMC (day 3 of lentiviruses infection) were serum starved for 48h (0.2% FBS). Total RNA was extracted from cells with TRIzol reagent (Life Technologies, Carlsbad, CA, USA) according to the manufacturer’s instructions. The specific mRNA quantification using RT-qPCR was performed by the genomic platform at the IRIC (Genomics Core Facility, Université de Montréal, Montreal, QC, Canada).

### Cell counting, cell viability and MTT assays

Equal number (4 X 10^5^) of VSMC infected with scrambled or ARF6 shRNA lentiviruses (day 3 of infection) were reseeded and serum starved for 48h (0.5%FBS). Cells were then stimulated or not with Ang II (100 nM) for 24, 48 or 72h. For each indicated time point, cells were stained using trypan blue, and live cells were manually counted using a hemocytometer. Cell proliferation was also measured by 3-(4,5-dimethylthiazol-2-yl)-2,5-diphenyl tetrazolium bromide (MTT) assay. VSMC were cultured in 96-well plates (3 X 10^3^ cells/well), serum starved for 48h (0.5% FBS) and then stimulated or not with Ang II (100 nM). After 3 days, 25 μl of MTT (5 mg/ml) was added to the culture medium and cells were incubated for an additional 2h at 37°C before being solubilized in 20% SDS/ 50% dimethylformamide solution overnight. Absorbance was measured at 570 nm with a reference wavelength at 450 nm using the microplate reader (Wallac Victor; Perkin Elmer, MA, USA). For ARF6 knock down experiments, VSMC were seeded in the 96-well plates at the third day of infection with scrambled or ARF6 shRNA lentiviruses. DMSO vehicle, DPI, AG1478 or ML171 was added to medium 8h before Ang II stimulation. For Rac1T^17^N overexpression experiments, cells were cultured in 96-well plate 24h after plasmids transfection.

### Statistical Analysis

Statistical analysis was performed using *t* test, one-way or two-way analysis of variance followed by a Bonferroni's multiple comparison test using GraphPad Prism (version 5, San Diego, CA, USA).

## Results

### ARF6 controls Ang II-induced Rac1 activation

**Fig 1:**
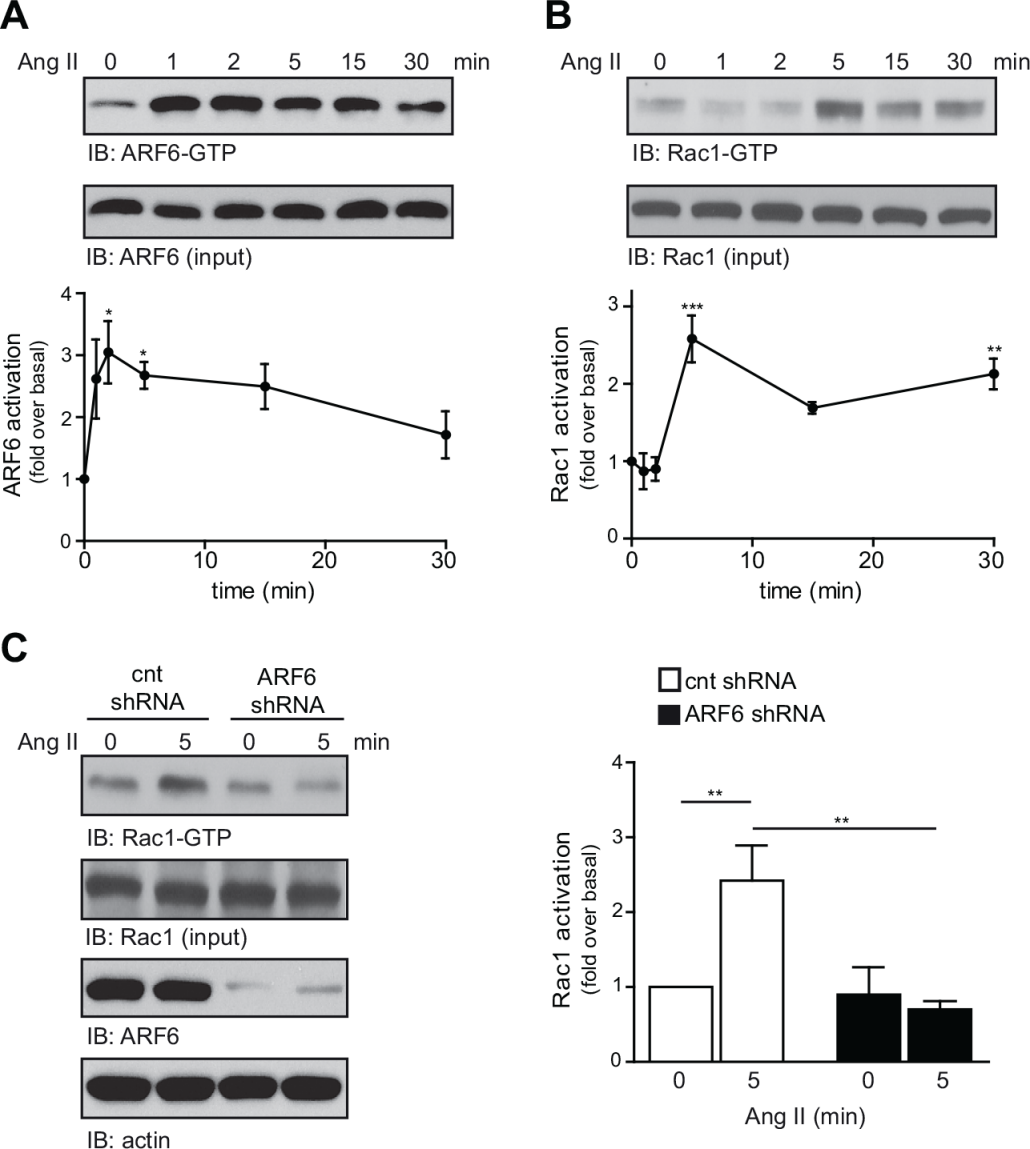
ARF6 controls Ang II-induced Rac1 activation. VSMC were stimulated for the indicated times with Ang II (100 nM) then lysed. (A). Endogenous levels of activated ARF6 (ARF6-GTP) captured by GST pulldown assay and total ARF6 (input) were assessed by Western blot analysis (n = 4, *P < 0.05). (B). Activated and total Rac1 levels were also determined by Western blot analysis (n = 3, **P < 0.01, ***P< 0.001). (C). VSMC were infected with scrambled or ARF6 shRNA lentiviruses. At the third day of infection, cells were serum starved for 48h and stimulated or not with Ang II for 5 min. Activated Rac1 was assessed as in (B). Quantifications are presented as fold-change over basal (cnt shRNA, t = 0) and are normalized to total protein content (n = 3, **P < 0.01).

First, we examined whether Ang II stimulation could promote the activation of ARF6 in rat aortic VSMC, a cellular model expressing endogenously the AT_1_R. As illustrated in Fig 1A, Ang II (100 nM) rapidly induced GTP-loading of ARF6 where maximal levels were observed within 2 min of stimulation (~ 3.1 fold). High levels of the activated GTPase remained sustained for 15 min, but slowly decreased afterwards (~1.7 fold; after 30 min). In addition, Ang II treatment of the cells also resulted in the activation of Rac1 (Fig 1B). Interestingly, the time-course of activation of the two small GTP-binding proteins was different. Maximal Rac1-GTP levels occurred after 5 min (~2.5-fold), slightly decreased after 15 min, but remained sustained for 30 min (~2.1 fold). The rapid GTP-loading of ARF6 suggests that activation of this GTPase might be an upstream event regulating Rac1 activation. We therefore next examined whether depleting endogenously expressed ARF6, in VSMC, could alter the ability of Ang II to promote GTP-loading of Rac1. As shown in Fig 1C, depletion of ARF6 had no effect on the basal level of activated Rac1, but completely abolished Ang II-mediated Rac1 activation after 5 min of stimulation. These results therefore suggest that Ang II-induced Rac1 activation is a process that is dependent upon the GTPase ARF6.

### Ang II-induced superoxide anion production is ARF6-dependent

**Fig 2:**
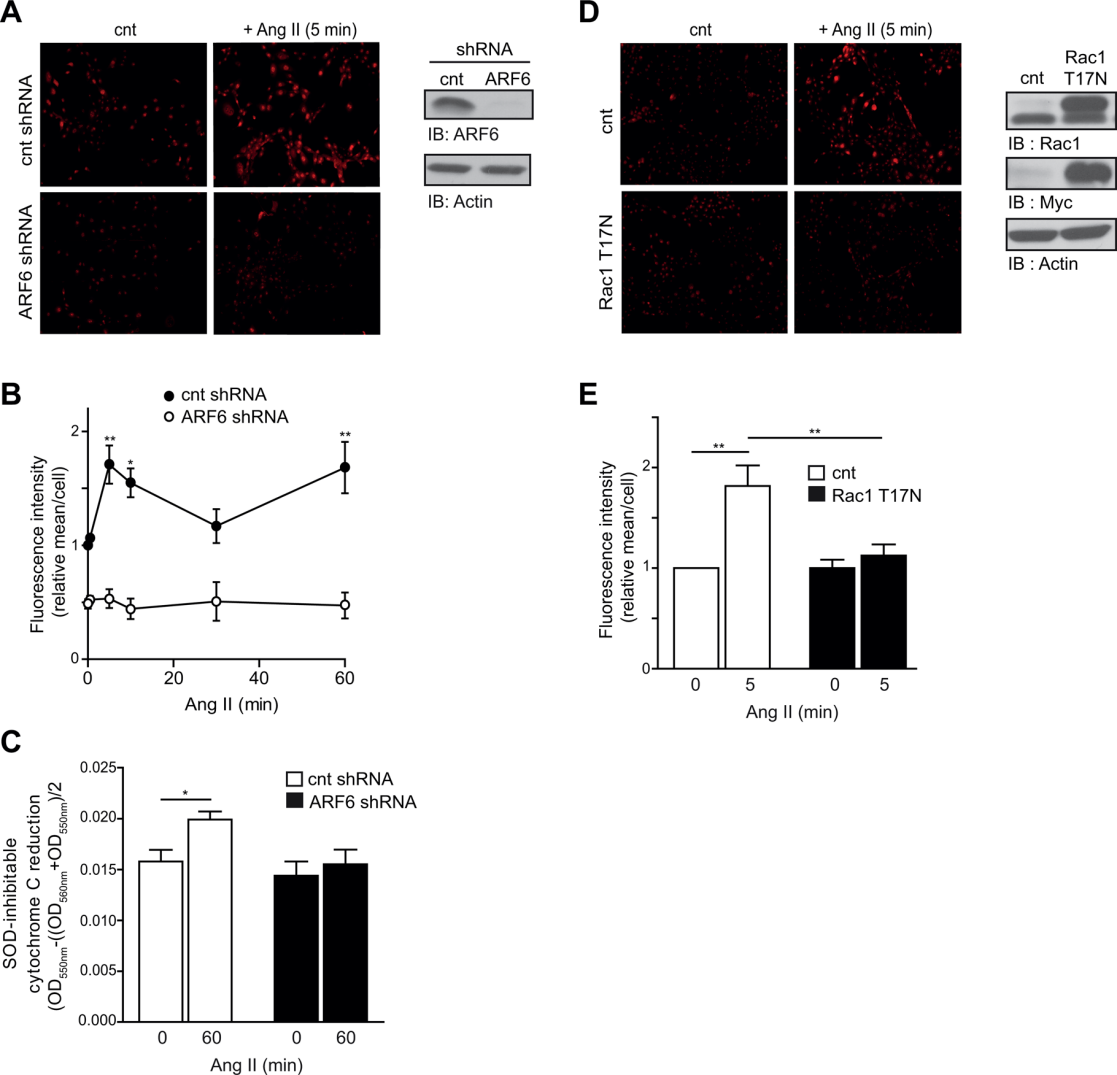
Ang II-induced superoxide anion production is ARF6 dependent. (A) Control and ARF6 depleted VSMC were stimulated with Ang II (100 nM) for the indicated time and incubated with DHE. Images are representative of each condition. Cells were examined to confirm ARF6 knock down by Western blot. (B) Graph represents quantitative analysis of fluorescence intensity mean per cell presented as fold change over basal (scrambled shRNA, t = 0). 3–4 images representative of 1000–1500 cells per time point of Ang II stimulation and per condition were analyzed using ImageJ software. DHE fluorescence were quantified and normalized to cells number following DAPI staining (n = 3, *P < 0.05, **P < 0.01). (C) Quiescent control and ARF6 depleted VSMC were stimulated or not with AngII (100 nM, 60 min) and superoxide anion levels were measured using cytochrome C reduction assay as described in materials and methods (n = 3, *P < 0.05). (D) Control and Rac1 T^17^N overexpressing VSMC were stimulated or not with Ang II. Superoxide anion levels were then evaluated by DHE staining as in (A). Rac1 T^17^N overexpression was confirmed by Western blot analysis. (E) Results obtained in (D) were quantified by analysis of fluorescence intensity average per cell as in (B) (n = 3, **P < 0.01).

It was previously reported that Ang II-induced superoxide anion production is dependent upon the activation of Rac1[15]. We therefore hypothesized that ARF6 may play a key role in controlling ROS production when VSMC are stimulated by Ang II. Here, we measured levels of superoxide anion using dihydroethidium (DHE) staining. In control VSMC, agonist-treatment significantly increased superoxide anion production in a biphasic fashion with two peaks: the first one after 5 min of stimulation (~1.7 fold) and the second one, after 1h (~1.68 fold) (Fig 2A and 2B). When cells were depleted of ARF6, we observed a significant decrease (~48%) in the basal level of superoxide anion present in VSMC, and interestingly ARF6 knock down totally prevented Ang II-induced increase of superoxide anion production (Fig 2A and 2B). To confirm these data, we used the cytochrome C assay as an alternative approach to measure ROS production [32]. As illustrated in Fig 2C, Ang II treatment significantly increased ROS levels. This effect was markedly impaired when cells were depleted of ARF6.

We next confirmed the role of Rac1 activation in Ang II-induced ROS production in our cells by overexpressing an empty vector (cnt) or the myc-tagged dominant negative Rac1T^17^N (myc-Rac1T^17^N) mutant. Myc-Rac1T^17^N overexpression did not affect the basal level of superoxide anions, but significantly reduced Ang II-promoted ROS increase after 5 min of stimulation (Fig 2D and 2E). Western blot assays were carried out in parallel to confirm expression of the Rac1 dominant negative mutant. These findings indicate that ARF6 plays a role in maintaining basal ROS level in VSMC and demonstrate that Ang II-induced superoxide anion production is dependent upon the subsequent activation of ARF6 and Rac1.

### ARF6 regulates Nox1 expression

**Fig 3:**
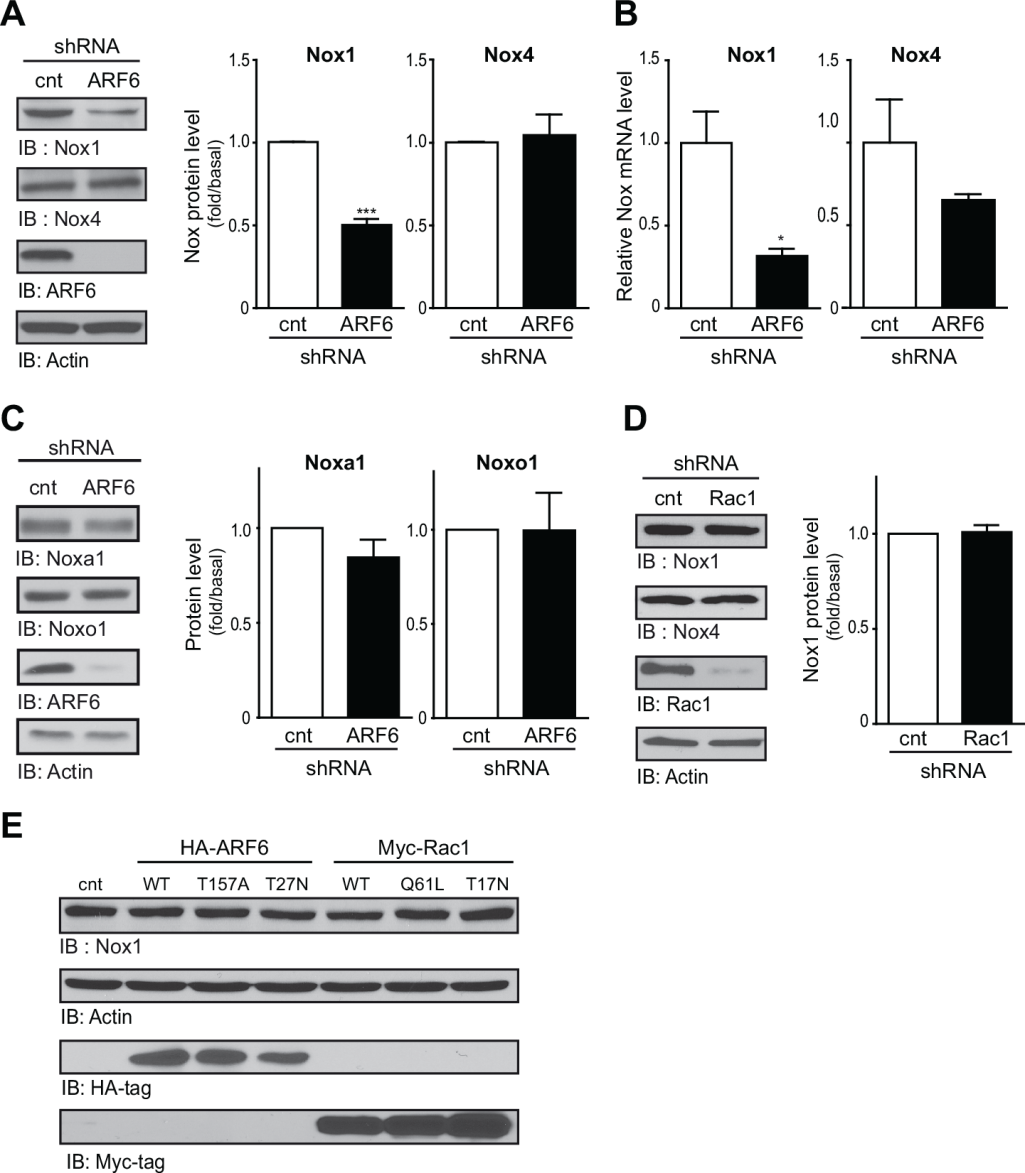
ARF6 regulates Nox1 expression. (A) Nox1 and Nox4 protein expression was examined in control and ARF6 depleted VSMC using Western blot analysis. Levels of ARF6 and actin were also determined. Graph represents quantification of all data (n = 3, ***P< 0.001). (B) mRNA levels of Nox1 and Nox4 were also assessed in cells infected with the control and ARF6 shRNA. Data were normalized to two control mRNA (GADPH and 4-HPRT) and presented as fold change over one control experiment (n = 3, *P < 0.05). (C) Noxa1 and Noxo1 protein levels were measured in control and ARF6 depleted VSMC using Western blot analysis. Graph represents quantification of three independent experiments (n = 3). (D) Nox1 and Nox4 protein expression was also examined in control and Rac1 depleted VSMC as in (A) (n = 3). (E) VSMC were transiently transfected with empty vector, HA-ARF6, HA-ARF6 T^157^A, HA-ARF6 T^27^N, myc-Rac1, myc-Rac1 Q^61^L or myc-Rac1 T^17^N and Nox1, actin, HA-tag and myc-tag levels were detected using Western blot analysis (n = 3).

Since depletion of ARF6 reduced the basal level of superoxide anions without affecting the basal Rac1 activation, we asked whether this ARF protein could control ROS levels by another mechanism, independent of Rac1. We therefore next sought to examine the role this ARF isoform may play on the expression of the major ROS generating enzymes, the NADPH oxidases. We first examined the expression profile of Nox isoforms in our cells. In VSMC, Nox1 and Nox4 were highly expressed (Fig 3A), while Nox2 was barely detectable (data not shown). Next, we evaluated protein and mRNA levels of Nox1 and Nox4 in control and ARF6-depleted VSMC, maintained in a quiescent state to avoid any exogenous regulation of Nox expression. As illustrated in Fig 3A, ARF6 depletion resulted in a ~ 50% decrease in Nox1 protein level and a ~ 68% decrease in mRNA level (Fig 3B). In contrast, knock down of the GTPase did not significantly affect Nox4 protein and mRNA levels. Similarly, expression of the regulatory subunits Noxa1 and Noxo1 remained unaffected in ARF6-depleted cells (Fig 3C).

To further understand how ARF6 could regulate expression of this ROS-generating enzyme, we first examined the role of Rac1. When endogenous expression of this Rac isoform was inhibited, expression of Nox1 remained the same in VSMC (Fig 3D) suggesting that Rac1 is not an intermediate in the regulation of the expression of this key enzyme. To further investigate the role of ARF6 and Rac1, we used a different strategy and overexpressed either the active or inactive mutant form of this small G protein. As illustrated in Fig 3E, expression of either wild type (WT), the fast cycling ARF6 mutant (ARF6T^157^A), or the dominant negative (ARF6T^27^N) form, remained ineffective in modulating Nox1 expression. Similarly, expression of Rac1, the constitutively active (Rac1Q^61^L), or the dominant negative (Rac1T^17^N) mutants had no effect on Nox1 expression (Fig 3E).

Together, these results demonstrate that the expression of ARF6 is essential for maintaining Nox1 expression levels in VSMC. This mechanism is independent of its activation state or of its ability to signal through Rac1.

### ARF6 mediates the ROS-dependent EGFR transactivation

**Fig 4:**
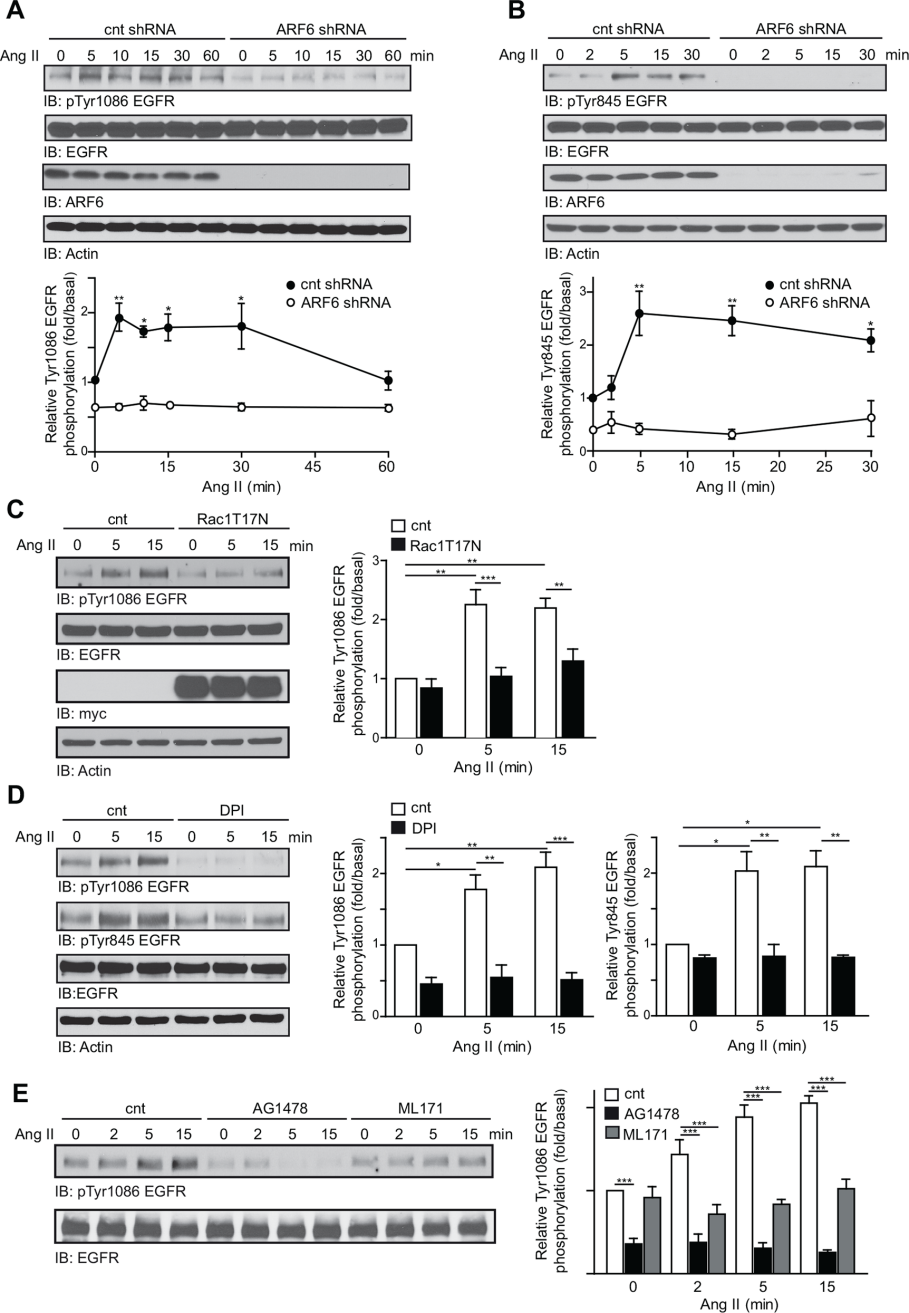
ARF6 mediates ROS-dependent EGFR transactivation. (A, B) Control and ARF6 depleted VSMC were stimulated with Ang II (100 nM) for the indicated times. EGFR phosphorylation levels on Tyr^1086^ (A) and Tyr^845^ (B) were assessed by Western blot analysis using specific phospho antibodies and data normalized to total EGFR (n = 3, *P < 0.05, **P < 0.01). (C) Control and Rac1 T^17^N overexpressing VSMC were stimulated or not with Ang II for the indicated times and EGFR phosphorylation levels on Tyr1086 were determined by Western blot analysis (n = 3, **P < 0.01, ***P < 0.001). (D) VSMC were pre-incubated with vehicle or DPI (10 μM) and stimulated with Ang II (100 nM) for the indicated times. EGFR phosphorylation levels on Tyr^1086^ and Tyr^845^ were determined as in (A, B). Data are the mean ± SEM of three independent experiments (n = 3, *P < 0.05, **P < 0.01, ***P < 0.001). (E) VSMC were pre-incubated with vehicle, AG1478 (100nM) or ML171 (5 μM) for 30 min then stimulated with Ang II (100 nM) for the indicated times. EGFR phosphorylation levels on Tyr^1086^ were measured as in (A) (n = 3,***P < 0.001).

To further define the molecular mechanisms by which ARF6 may regulate Ang II-mediated function in VSMC, we examined the involvement of the EGFR. Previous reports have demonstrated that ROS acts as a signaling molecule to induce EGFR transactivation [16]. We therefore next assessed Ang II-mediated EGFR transactivation in control (scrambled shRNA) and ARF6-depleted (ARF6 shRNA) cells by assessing the phosphorylation state of two different tyrosine sites on the EGFR intracellular domain, Tyr^1086^ and Tyr^845^. As shown in Fig 4A and 4B, Ang II similarly increased the phosphorylation of both tyrosine residues in control cells. EGFR activation reached its maximum at 5 min (~1.9 fold for Tyr^1086^ and ~2.6 fold for Tyr^845^) and was sustained for 30 min. In contrast, ARF6 depleted cells showed a decrease in basal EGFR activation (~ 37% less for Tyr^1086^ and ~60% less for Tyr^845^) and Ang II-induced phosphorylation of Tyr^1086^/Tyr^845^ was suppressed suggesting that ARF6 is required for transactivation of this RTK by the AT_1_R. When cells expressed the dominant negative mutant of Rac1, phosphorylation of the EGFR was also reduced (Fig 4C). To further investigate the role of ROS in this process, we examined Ang II-induced EGFR transactivation in control cells (DMSO) and cells treated with diphenyliodonium (DPI 10 μM), a broad spectrum flavoprotein inhibitor. Interestingly, we found that blocking Nox enzymes by DPI completely inhibited the Ang II-mediated phosphorylation of Tyr^1086^ and Tyr^845^ on the EGFR (Fig 4D). The role of Nox1 was confirmed when we treated cells with ML171 (Fig 4E).

### ARF6 is required for the activation of ROS sensitive MAPK and proliferation

**Fig 5:**
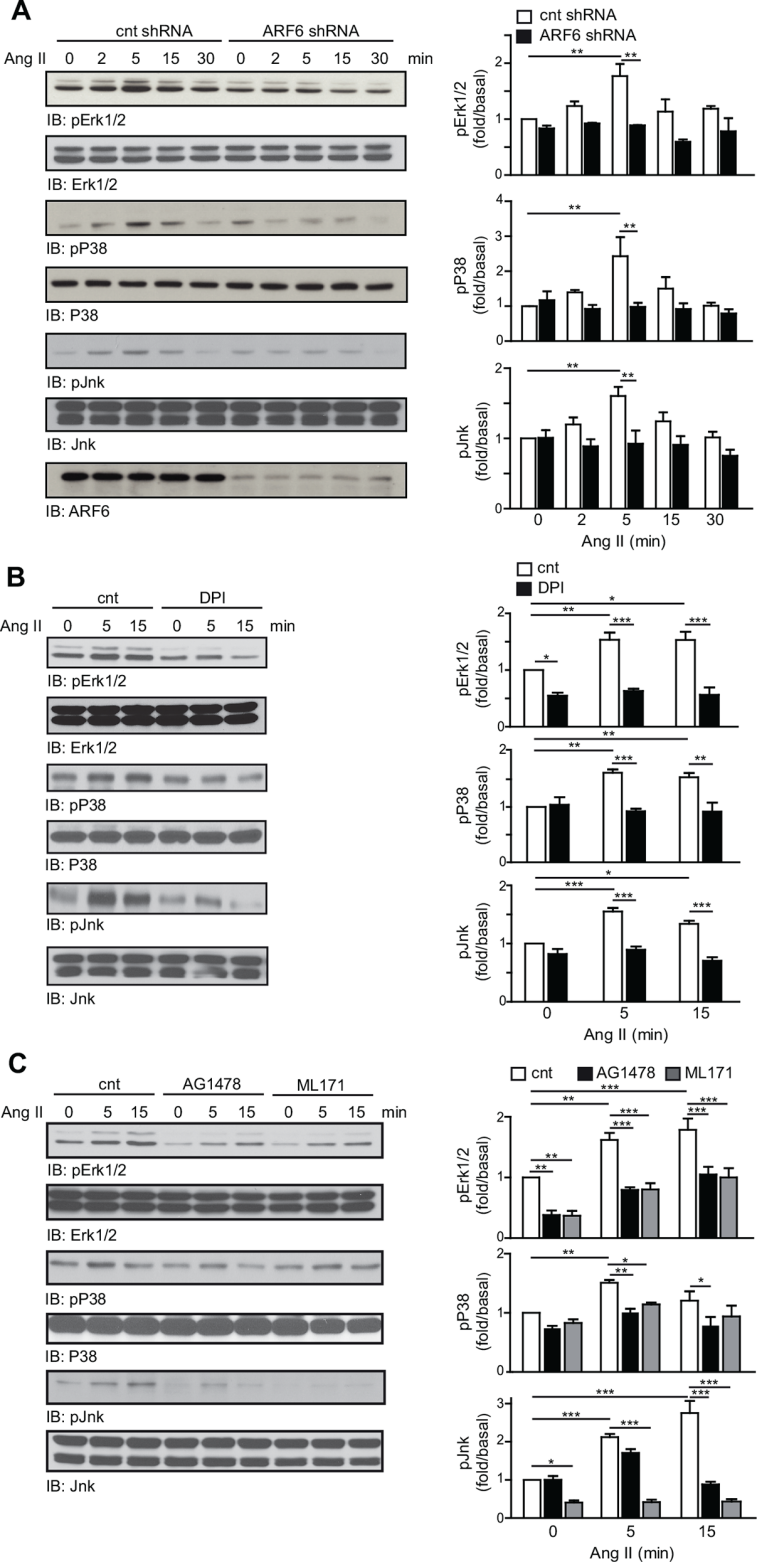
ARF6 is required for the activation of the ROS sensitive Erk1/2, p38 and Jnk. (A) Control and ARF6 depleted VSMC were stimulated with Ang II (100 nM) for the indicated times. Phosphorylation and total levels of Erk1/2, p38, and Jnk were examined (n = 3, **P < 0.01). (B) Cells were treated with vehicle or DPI (10 μM) and stimulated with Ang II (100 nM) for the indicated times. Phosphorylation levels of Erk1/2, p38 and Jnk were assessed as in (A). Results are representative of three independent experiments and quantifications are the mean ± SEM (n = 3, *P < 0.05, **P < 0.01, ***P < 0.001). (C) Cells were treated with vehicle, AG1478 (100 nM) or ML171 (5 μM) for 30 min then stimulated with Ang II for the indicated times. Phosphorylation and total levels of Erk1/2, p38, and Jnk were assessed by Western blot analysis (n = 3, *P < 0.05, **P < 0.01, ***P < 0.001).

To further define the role of the AT_1_R/ARF6/Rac1/ROS/EGFR signaling axis, we examined activation of mitogenic signaling cascades. We first assessed the phosphorylation state of Erk1/2, p38 and Jnk. In control cells, Ang II increased the activation of all three MAPK. Time-course profiles were similar with a peak after 5 min of Ang II treatment (~1.8 fold for Erk1/2, ~2.4 fold for p38 and ~1.6 fold for Jnk) (Fig 5A). However, depletion of ARF6 markedly blocked the Ang II-induced phosphorylation of Erk1/2, p38 and Jnk (Fig 5A). As previous studies have reported that ROS is a key regulator of MAPK activation [17, 18, 20], we next confirmed their role in our cells. VSMC were treated with either a vehicle or the NADPH oxidase inhibitor, DPI, for 30 min and subsequently stimulated with Ang II. As illustrated in Fig 5B, inhibition of ROS production completely prevented activation of the three MAPK upon Ang II stimulation. Specific inhibition of EGFR phosphorylation with AG1478 or Nox1 with ML171 resulted in the same effect (Fig 5C). These findings and the results above further support our hypothesis that ARF6 controls ROS production to mediate Ang II-promoted MAPK activation in VSMC.

**Fig 6:**
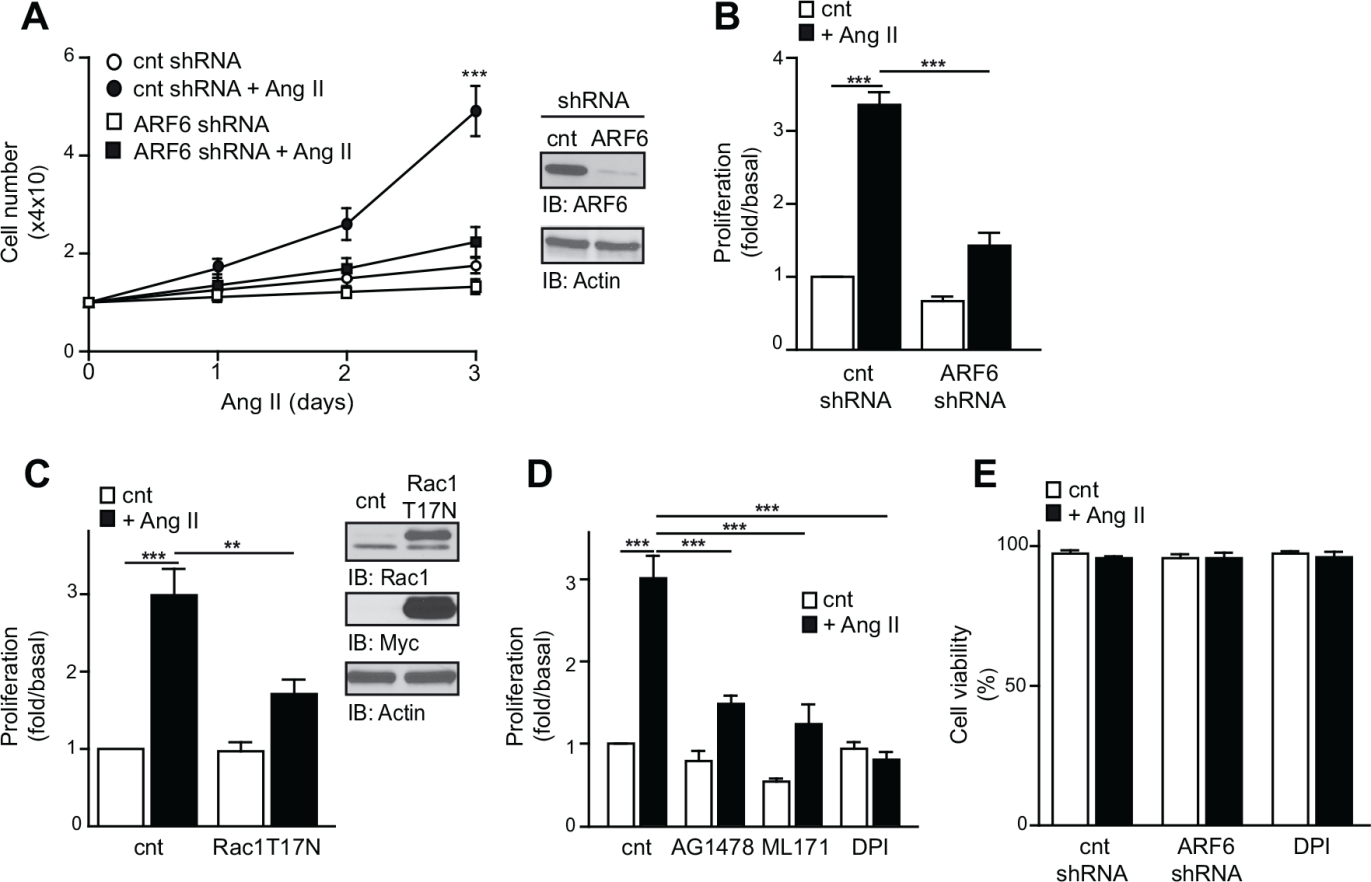
ARF6 mediates Ang II induced cellular proliferation through ROS. (A) Control and ARF6 depleted cells were stimulated or not with Ang II (100 nM) for the indicated times. Manual cell count was performed for each experimental condition (n = 3, ***P < 0.001). (B) Proliferation of control and ARF6 depleted cells stimulated or not with Ang II was assessed using the MTT assay (n = 3, ***P < 0.001). (C) Control and Rac1 T^17^N expressing cells were left untreated or stimulated with Ang II (100 nM) for 72h. Proliferation was assessed using the MTT assay as in (B) (n = 3, **P < 0.01, ***P < 0.001). (D) Cells incubated with DMSO, AG1478 (100 nM), ML171 (5 μM) or DPI (1 μM) were stimulated or not with Ang II for 72h and proliferation was assessed using MTT assay (n = 3, ***P < 0.001). (E). Cellular viability of control, ARF6 depleted and DPI treated cells stimulated or not with Ang II was determined using trypan blue (n = 3)

We next examined a key Ang II-mediated cellular response, proliferation. As illustrated in Fig 6A, depletion of ARF6 markedly impaired the ability of VSMC to proliferate upon Ang II treatment. After 3 days of agonist stimulation, cell number was enhanced by ~ 4.9-fold in the control, and ~ 1.7-fold in the ARF6-depleted conditions (Fig 6A). Similar results were obtained when we used an alternative approach to assess cell growth, the MTT assay (Fig 6B). As illustrated in Fig 6C, expression of the dominant negative Rac1T^27^N mutant effectively reduced Ang II-mediated VSMC proliferation (43%). To confirm that this cellular response was indeed dependent upon ROS generation, we tested the effect of DPI and ML171. Interestingly, Ang II-induced VSMC proliferation was markedly inhibited when cells were incubated with these inhibitors (Fig 6D). Blockade of EGFR activation by AG1478 also markedly reduced proliferation (Fig 6D). Next, we performed cell viability assays to verify whether the reduced proliferation that we observed in ARF6-depleted cells could be a result of increased cell death. We found that neither ARF6 knock down nor Nox enzyme blocking affected cell viability (Fig 6E). Altogether, our findings show that ARF6 acts through ROS to promote Ang II-dependent VSMC proliferation.

## Discussion

Our findings provide evidence of a completely new role for ARF6, classically known as a regulator of endocytic membrane trafficking, actin remodelling and phospholipids metabolism [22]. Here, we demonstrate that this GTPase is a key regulator of Ang II-induced ROS production by a molecular mechanism involving the regulation of Rac1 activation as well as Nox1 protein expression. Once ROS levels are enhanced in cells, these signaling intermediates regulate MAPK activation through transactivation of the EGFR and ultimately VSMC proliferation.

Previous studies have shown a coordinated action between ARF6 and Rac1 in different cellular models [28–31]. We have previously demonstrated that the two GTPases can directly associate upon Ang II stimulation [24]. Here, we report that Ang II activates both endogenous ARF6 and Rac1 in VSMC. Interestingly, we observed that ARF6 activation occurred earlier than Rac1 suggesting that GTP-loading of these two GTPases may be sequential. This hypothesis was confirmed by the loss of Ang II-induced Rac1 activation when ARF6 was depleted. Different possible molecular mechanisms have been proposed for ARF6-mediated activation of Rac1. Santy and colleagues reported that this ARF isoform modulated Rac1 activity through the regulation of the Dock180/Elmo complex in MDCK cells [33]. Furthermore, ARF6 was also shown to promote Rac1 activation by controlling the recruitment of a protein complex containing the ARFGAP GIT1/2 and the RacGEF β-PIX [34, 35]. Interestingly, it was proposed that ARF6 could regulate membrane targeting of Rac1 necessary for its activation through transformation of lipid rafts [36]. Whether, in VSMC, ARF6 regulates the function of a RacGEF remains to be determined.

The role of Rac1 has been extensively studied in the context of ROS production. This GTP-binding protein was defined as an essential component of Nox1, Nox2 and Nox3 complexes [15]. In addition to controlling the Ang II-mediated activation of Rac1, we observed that ARF6 knock down resulted in a substantial decrease of basal levels of superoxide anions. We demonstrate that this is due to a direct effect of the GTPase on Nox1 expression specifically, although aortic rodent VSMC expresses both Nox1 and Nox4. Our results suggest that the presence of ARF6, rather than its activation state, controls Nox1 expression. Interestingly, it was previously reported that expression of this Nox isoform was under the control of the transcription factors GATA-4,-5 and -6 in colon epithelial cells [37]. In VSMC, GATA-6 has been shown to be a key regulator of gene expression [38]. Whether ARF6 regulates Nox1 expression through this specific transcription factor needs to be further investigated.

EGFR transactivation plays an important role in Ang II signaling. In VSMC, this process was shown to require activation of metalloprotease 2/9 and Src [39]. Our findings further demonstrate that Ang II-mediated transactivation of the EGFR requires ARF6 since knock down of this GTPase can prevent phosphorylation of Tyr^1086^ and Tyr^845^ on the EGFR. Interestingly, the later residue is a target of Src, which activation was reported to be sensitive to ROS [40]. We demonstrate here that pre-treatment of cells with the specific Nox inhibitor, DPI, prevented Ang II-induced EGFR transactivation. Mifune and colleagues have reported that ROS production occurs upstream of EGFR transactivation upon Ang II stimulation [16]. In contrast, others proposed that EGFR transactivation occurred upstream and was required for ROS production [13]. In our cell model, we suggest that ARF6-mediated ROS production is required for EGFR transactivation. These data do not however exclude the possibility that transactivation of EGFR may lead to an additional production of ROS. Furthermore, we found that Ang II-induced activation of the MAPK Erk1/2, p38 and Jnk was completely suppressed in ARF6-depleted cells. These signaling events were also dependent on ROS production since the activation of all of these three MAPK was blocked by inhibiting Nox enzymes confirming their redox sensitivity. Although the sensitivity of p38 and Jnk to ROS has been previously reported [17, 18, 41, 42], the sensitivity of Erk1/2 to ROS remains controversial [18–21, 42]. Our results support the observation that ARF6-dependent ROS generation is a key step for Ang II-dependent MAPK activation. Because MAPK are important for cellular proliferation, we therefore expected that ARF6 would play a crucial role in this biological response. Our results indicate that this GTPase indeed controls basal as well as Ang II-dependent VSMC proliferation. This first observation may be explained by the low level of ROS found in ARF6-depleted cells. Although ARF6 knock down completely abolished superoxide anion production as well as EGFR transactivation and MAPK activation induced by Ang II, we observed that depletion of ARF6 only partially inhibited VSMC proliferation. However, inhibition of Nox activity by DPI was more effective in preventing Ang II-mediated cell proliferation. The more potent effect of this chemical inhibitor may depend on its ability to efficiently inhibit all Nox isoforms compared to ARF6, which may be selective for Nox1.

**Fig 7:**
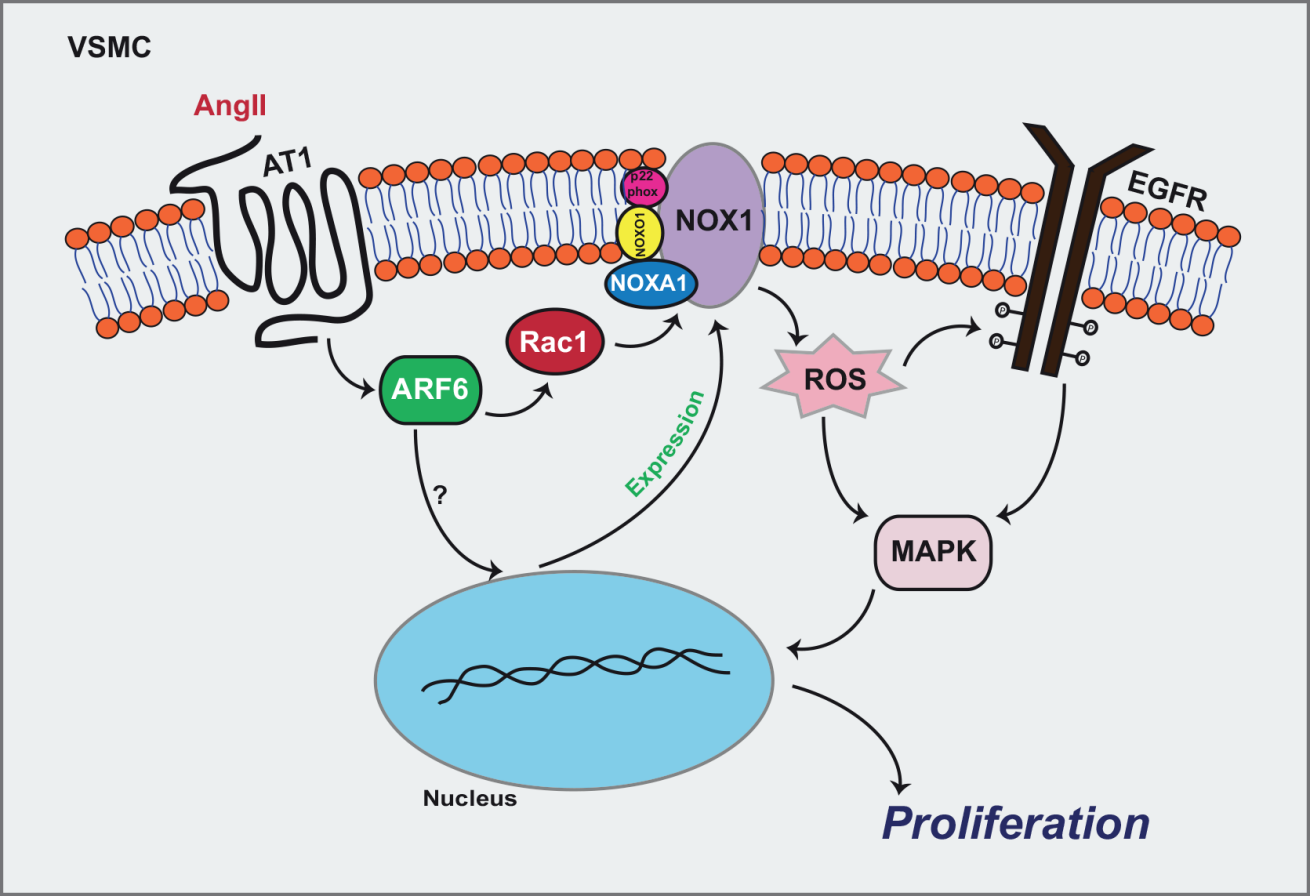
Schematic representation of the molecular mechanism by which ARF6 mediates Ang II promoted ROS generation and proliferation of VSMC. Stimulation of the AT_1_R by Ang II leads to the activation of ARF6, which is essential for the activation of Rac1. This Rho GTPase acts to control NADPH oxidase and formation of ROS. These signaling intermediates play numerous roles in VSMC. They are essential for EGFR transactivation and MAPK phosphorylation. In addition, ARF6 can regulate Nox1 expression to further support ROS production. Altogether, our findings show that ARF6 is a molecular switch regulating cellular proliferation.

In sum, our study supports a new role for the GTPase ARF6 in ROS production and Ang II-induced VSMC proliferation. We demonstrate that ARF6 promotes ROS production by a mechanism involving the activation of Rac1 and Nox1 expression. Through ROS, ARF6 mediates EGFR transactivation and MAPK activation leading to VSMC proliferation (Fig 7). How ARF6 regulates Nox1 expression and whether ARF6 is implicated in the regulation of other proliferation pathways remains to be defined. Because of the key role ROS plays in cardiovascular diseases, ARF6 could be a new potential therapeutic target for the treatment of atherosclerosis, restenosis after angioplasty and hypertension.
